# Interface induce growth of intermediate layer for bandgap engineering insights into photoelectrochemical water splitting

**DOI:** 10.1038/srep27241

**Published:** 2016-06-02

**Authors:** Jian Zhang, Qiaoxia Zhang, Lianhui Wang, Xing’ao Li, Wei Huang

**Affiliations:** 1 Key Laboratory for Organic Electronics and Information Displays & Institute of Advanced Materials (IAM), Jiangsu National Synergetic Innovation Center for Advanced Materials (SICAM), Nanjing University of Posts & Telecommunications, Nanjing 210023, China; 2 Key Laboratory of Flexible Electronics (KLOFE) & Institute of Advanced Materials (IAM), Jiangsu National Synergetic Innovation Center for Advanced Materials (SICAM), Nanjing Tech University (NanjingTech), Nanjing 211816, China

## Abstract

A model of interface induction for interlayer growing is proposed for bandgap engineering insights into photocatalysis. In the interface of CdS/ZnS core/shell nanorods, a lamellar solid solution intermediate with uniform thickness and high crystallinity was formed under interface induction process. Merged the novel charge carrier transfer layer, the photocurrent of the core/shell/shell nanorod (css-NR) array was significantly improved to 14.0 mA cm^−2^ at 0.0 V vs. SCE, nearly 8 times higher than that of the perfect CdS counterpart and incident photon to electron conversion efficiency (IPCE) values above 50% under AM 1.5G irradiation. In addition, this array photoelectrode showed excellent photocatalytic stability over 6000 s. These results suggest that the CdS/Zn_1−x_Cd_x_S/ZnS css-NR array photoelectrode provides a scalable charge carrier transfer channel, as well as durability, and therefore is promising to be a large-area nanostructured CdS-based photoanodes in photoelectrochemical (PEC) water splitting system.

Photoelectrochemical (PEC) water splitting for hydrogen generation has attracted great attention since the process was first reported^1^. Under solar light irradiation, electron-hole pairs are excited in the depletion region of a typical semiconductor electrode and then transfer to the surface of electrode to be consumed (reduction: H^+^ + e → H_2_ and oxidation: H_2_O + h → O_2_)^2^. As one of the powerful strategies for promoting charge directional transport and light harvesting efficiency, one-dimensional semiconductor arrays with uniform shapes and morphologies, such as nanowires^3^,^4^, nanorods^5^,^6^, and nanotubes^7^,^8^ have been widely studied as photoelectrodes in this process. Besides, bandgap engineering provide the possibility to further improve the solar energy conversion efficiency by stacking layers of semiconductors with precise thicknesses and sequences design based on the superb architectures above^9^,^10^. In addition, designing and coating the lattice-matched semiconductors on the original catalyst as charge carrier transfer layer can realize nearly defect-free interfaces to protect the unstable semiconductor from passivation and photocorrosion^11^.

As a classical and vital II-VI semiconductor, n-type CdS has been employed as an efficient photoanode over the last thirty years since the suitable flat-band potential (−0.9 V vs. NHE) and strong light absorption capacity^12^,^13^,^14^. However, the inherent drawbacks of CdS such as the limited carrier life time, high electron-hole recombination rate, and especially photocorrosion have limited the performance of solar energy conversion^15^. One-dimensional nanoarchitectures as well as bandgap engineering are also effective approachs to overcome the above-mentioned disadvantages. Consequently, the research on one-dimensional CdS nanostructures has received worldwide concerns and led to notable increase of related research reports^16^. Based on the one-dimensional CdS nanostructures from templates, substrates, and seeds, various materials with matched bandgap including TiO_2_^17^, g-C_3_N_4_^18^, CdSe^19^, Cu_2_S^20^, and ZnFe_2_O_4_^21^ have been merged into CdS nanostructures to form heterojunctions to realize efficient solar energy conversion. In particular, Ji *et al*. proposed a strategy based on the bandgap engineering in PEC cell and reported the highest photocurrent value (35 mA cm^−2^, 100 mW cm^−2^) for a silicon-based photocathode. The lattice well matched lamellar SrTiO_3_ (4-nm-thick) growing on the surface of silicon by molecular beam epitaxy can act as a protection layer, as well as a tunneling junction for charge transportation due to the low density of interface defects^22^. Very recently, we synthesized a dense array of CdS/ZnS core/shell nanorods film through a simple two-step aerosol assisted chemical vapor deposition (AACVD) method^23^. The as-synthesized CdS/ZnS photocathode displayed enhanced PEC performance with a photocurrent density of 7.8 mA cm^−2^ (0 V, vs. SCE) under AM 1.5G irradiation. However, inspired by advances above^22^, we realized that the thickness^24^ and lattice parameter^9^ of the ZnS shell limited the function of the tunneling junction layer.

In this work, we used a simple AACVD method to induce the growth of ultrathin layers of Zn_1−x_Cd_x_S (~12 nm) on the interface of CdS/ZnS core/shell nanorods. Two perfect interfaces with very low density of defect states were established at the same time. More important, the intermediate layer Zn_1−x_Cd_x_S with lattice well matched can act as a bridge to smooth the band gap between CdS and ZnS for the establishment of two low density of defect states of the interfaces in the unique one-dimensional core/shell arrays^22^. Thanks to the lamellar solid solution intermediate, the transfer efficiency of charge carriers can be enhanced effectively. The obtained CdS/Zn_1−x_Cd_x_S/ZnS core/shell/shell nanorod (css-NR) photoanodes achieved 14.0 mA cm^−2^ (0 V, vs. SCE) under AM 1.5G illumination from PEC water splitting, which is almost 8 times compared with the bare CdS nanorods array electrode. The growth of ultrathin intermediate layer by interface induction will advance the utilization of different core/shell nanomaterials in the PEC water splitting applications.

## Results

**Figure 1:**
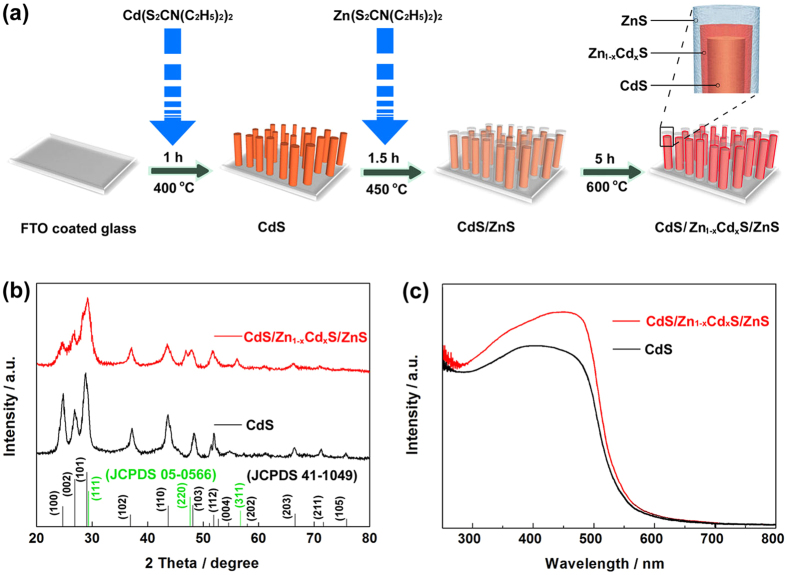
(**a**) Schematic of the deposition process for the CdS/Zn_1−x_Cd_x_S/ZnS css-NRs prepared on FTO coated glass. (**b**) XRD patterns of CdS and CdS/Zn_1−x_Cd_x_S/ZnS nanorods (scraped from the substrate of FTO glass). (**c**) UV-vis absorption spectra of CdS and CdS/Zn_1−x_Cd_x_S/ZnS nanorods.

Among the alternative electrode preparation processes, aerosol assisted chemical vapor deposition (AACVD) is a relatively convenient method that is easy to deposit high quality thin films electrode^25^. The fabrication of CdS/Zn_1−x_Cd_x_S/ZnS css-NR array electrode through this method is shown schematically in Fig. 1a. In the current work, two critical deposition parameters including the carrier gas flow rate and the substrate temperature are adjusted to guarantee homogeneous nucleation of the vaporized precursor (Cd(S_2_CN(C_2_H_5_)_2_)_2_) in the gas phase and growth on the substrates (FTO) in the first step. The CdS crystal nucleus then adsorbed on the surface of FTO, and a ripening/growing process leads to the formation of nanorods. Similarly, homogeneous nucleation can be implemented to insure ZnS crystal nucleus can adsorb on the surface of crystalline CdS nanorods uniformly. The ripening/growing temperature is maintained at 450 °C which is much lower than the crystallization temperature of ZnS crystal (520 °C) according to the previous research^23^. Finally, the most important, the solid solution Zn_1−x_Cd_x_S transition layer can be induced to grow through lattice matching in the interface of the CdS/ZnS during the temperature-rise process. Thanks to the lattice matched well each other, interface induction for intermediate layer growing is achieved after crystallization at 600 °C.

The phase structure of the as-deposited CdS and CdS/Zn_1−x_Cd_x_S/ZnS css nanorods were examined with the powder XRD technique (scraped from the substrate of FTO glass) as shown in Fig. 1b. The XRD pattern of the bare CdS sample is shown in the middle of Fig. 1b, in which all the diffraction peaks are indexed to the wurtzite structure of CdS (JCPDS card no. 41-1049) (bottom pattern in Fig. 1b). After merging of ZnS shell and growth of Zn_1−x_Cd_x_S intermediate layer, as shown in the top of Fig. 1b, three new diffraction peaks at 29.26° (111), 47.53° (220), and 56.72° (311) are observed clearly corresponding to the zinc blende ZnS shell (JCPDS card no. 05-0566). According to the previous study, coating another semiconductor shell with smaller lattice constant around the original core, a slight shift of the diffraction maxima (~2°) toward larger angles can be observed in the core/shell sample^23^,^26^,^27^. However, any shift of the diffraction maxima can be detected in the Fig. 1b due to the lattice well matched intermediate layer (Zn_1−x_Cd_x_S) which has the low density of defect states of the two interfaces. Figure 1c shows UV-vis absorption spectra of CdS and css nanorods. Bare CdS nanorods has an absorption edge at ~550 nm corresponding to the energy band gap value at about 2.35 eV. A slight red shift can be found in Fig. 1c (CdS/Zn_1−x_Cd_x_S/ZnS curve) after growing with Zn_1−x_Cd_x_S and ZnS shells because ZnS shell with ~80 nm thickness can weaken the leakage of excitons from the CdS core to the ZnS shell across the interlayer (Zn_1−x_Cd_x_S)^28^.

**Figure 2:**
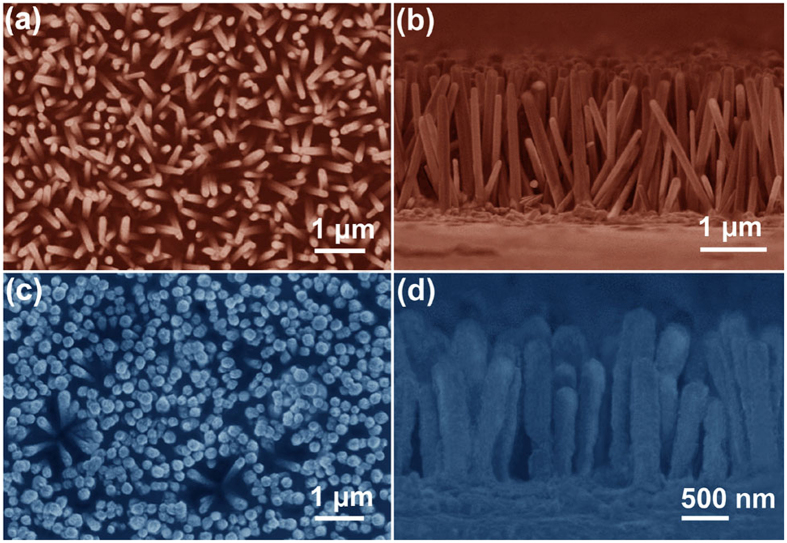
FESEM images of the CdS and CdS/Zn_1−x_Cd_x_S/ZnS css ranorod arrays. (**a,c**) Top and (**b**,**d**) cross-sectional view FESEM images of CdS and CdS/Zn_1−x_Cd_x_S/ZnS nanorod arrays.

After initial deposition, the typical FESEM images of CdS ranorod arrays that served as an ideal scaffold to support shells in the css-NR structures can be seen in Fig. 2a,b. Smooth and vertically aligned CdS nanorods grown directly on FTO substrates can be observed clearly with consistent diameter of ~200 nm and with lengths of ~1.5 μm through the comparing of the top and cross-sectional view images. Employing the identical deposition device used for the original CdS nanorod array film, ZnS shell with controllable thicknesses can be further deposited around the bare CdS nanorods to form core/shell nanorods. Figure 2c shows a typical morphologies of the CdS/Zn_1−x_Cd_x_S/ZnS css nanorods array prepared after once deposition (Zn(S_2_CN(C_2_H_5_)_2_)_2_) and twice thermal (450 and 600 °C) process. The cross-sectional image displays that the ZnS shell is uniformly coated along the entire length of the CdS nanorods with an average diameter up to ~350 nm from Fig. 2d.

**Figure 3:**
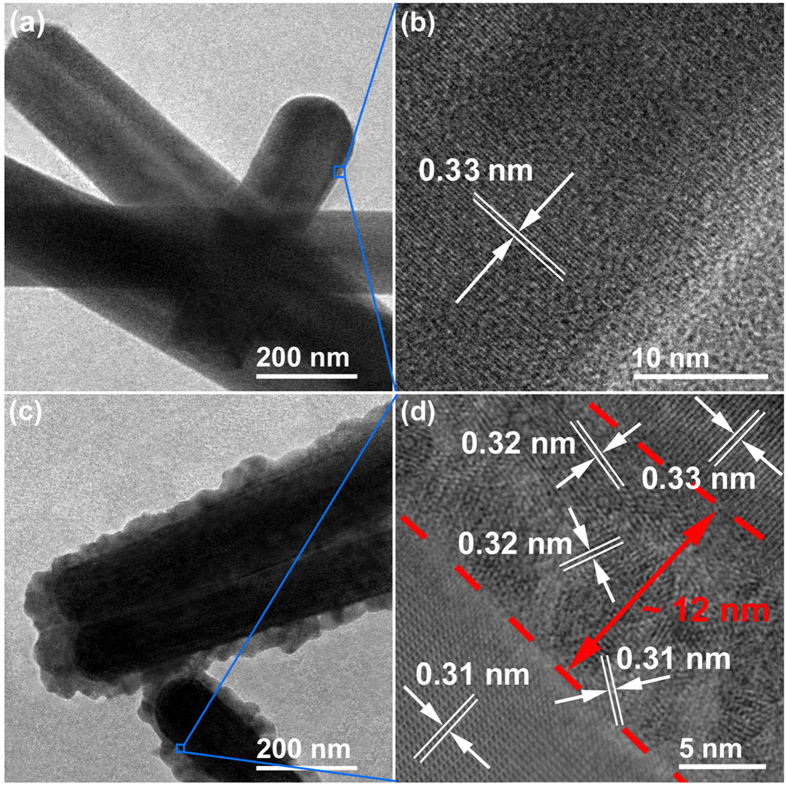
TEM and HRTEM images of CdS (**a,b**) nanorods and CdS/Zn_1−x_Cd_x_S/ZnS css nanorods (**c,d**).

Shells growth of ZnS and Zn_1−x_Cd_x_S around CdS nanorods are further confirmed by the TEM and HRTEM images as shown in Fig. 3. Consistent with the FESEM analysis, a clean surface of bare CdS nanorods with consistent diameter of ~200 nm is observed (Fig. 3a). From HRTEM image of the selected area shown by the blue square in Fig. 3a, the clear lattice spacing of CdS is 0.33 nm, which corresponds to the (002) plane of the wurtzite CdS^29^. For CdS/Zn_1−x_Cd_x_S/ZnS css-NRs, the entire surface of the CdS nanorods was uniformly coated with a ZnS shell (~80 nm in thickness), showing typical core/shell nanoarchitectures (Fig. 3c). To further verify the formation of lamellar solid solution intermediate, the selected area shown by the blue square in Fig. 3c was also observed by HRTEM (Fig. 3d). Two nearly defect-free interfaces are marked by the red dashed lines as shown in Fig. 3d. Beside two sets of clear lattice spacing (0.33 and 0.31 nm) corresponding to the (002) plane of the wurtzite CdS and (111) planes of the zinc blended ZnS, respectively^30^, several sets of lattice spacing of 0.31 and 3.2 nm with different orientations are detected in the interlayer of the css-NRs which caused by the passive diffusion (interface induction from CdS) of Zn ion to the CdS crystal to assemble Zn_1−x_Cd_x_S^31^,^32^,^33^ intermediate layer during the temperature-rise process. Figure S1 and 2 (Supporting Information) display the morphology of intermediate state (ZnS/CdS nanorods) which conforms to our expectation that Zn ion from amorphous ZnS shell can diffuse easily in the interface of ZnS/CdS without the imprisonment from ZnS crystal cell. The two perfect lattice matched interfaces created from lamellar solid solution intermediate can smooth the band barrier through nearly defect-free interfaces of the quasi-homojunction^9^. Based on the above analysis results, it can be concluded that high-quality CdS/Zn_1−x_Cd_x_S/ZnS css-NRs were successfully fabricated through this convenient, two-step AACVD process.

**Figure 4:**
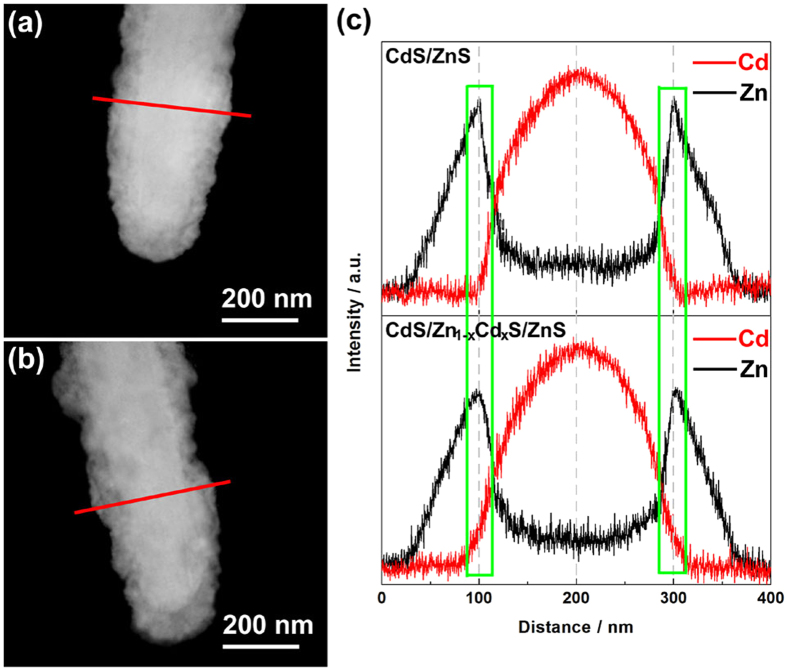
HAADF-STEM images of CdS/ZnS core/shell nanorod (**a**) and CdS/Zn_1−x_Cd_x_S/ZnS css-NR (**b**) with the corresponding EDS line scan measured along the diameter of the nanorods (marked by red lines) (**c**).

HAADF-STEM images of the cross-section and the corresponding EDS line scan analysis verify the spatial distribution of the compositional elements along the radial direction of nanorods as shown in Fig. 4. As observed from Fig. 4a,b, there is an intense contrast between the core and the shell, indicating core/shell structures. In the intensity profile of the compositional elements as shown in Fig. 4c, Cd are mainly confined within the core area of nanorod, while a higher intensity of Zn is found in the shell region. In particular, the subtle differences of the signals in the two EDS line scan profiles can be revealed through the comparing of the two selected areas shown by the green squares (Fig. 4c). The more moderate changing of the CdS/Zn_1−x_Cd_x_S/ZnS curve shown in the green squares can demonstrate the existence of intermediate layer and the thickness of the intermediate (~10 nm) accord with the result of HRTEM.

**Figure 5:**
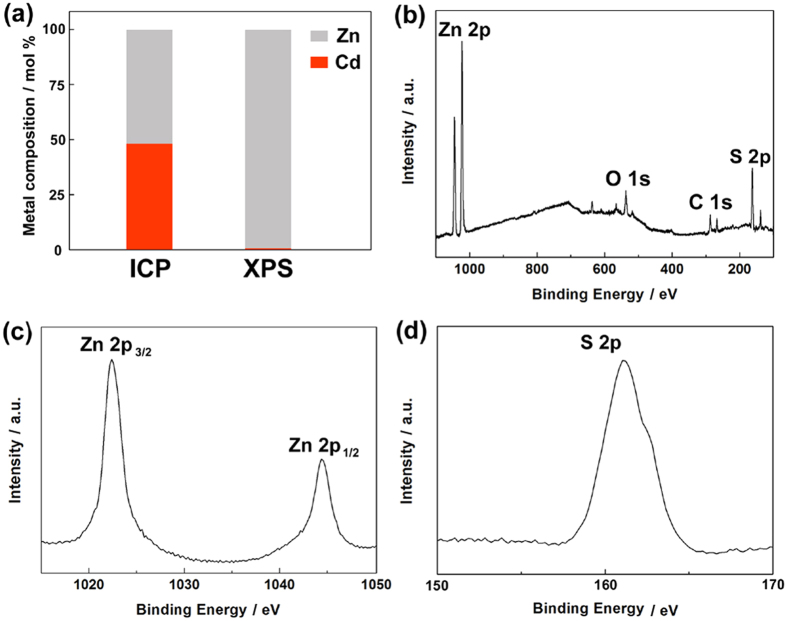
The bulk and surface metal compositions of CdS/Zn_1−x_Cd_x_S/ZnS sample measured by ICP (left) and XPS (right) methods, respectively (**a**). XPS survey spectrum (**b**), Zn 2p spectrum (**c**), and Cd 3d spectrum (**d**) of CdS/Zn_1−x_Cd_x_S/ZnS sample.

To get deeper insight into the bulk and surface compositions of the synthetic CdS/Zn_1−x_Cd_x_S/ZnS samples, ICP and XPS techniques were conducted. In the ICP result as shown in Fig. 5a, the Zn/Cd ratio is approximate 1:1 which close to the stoichiometric ratio of Zn and Cd in the initial precursor solution, whereas in the XPS result, the Zn/Cd ratio is approximate 99:1. The trace amounts of Cd can attribute to the exposure of the core while the sample was scraped from the substrate of FTO glass. This accuracy control is difficult to achieve by some other methods, such as hydrothermal and coprecipitation^34^,^35^. The survey scan spectrum (Fig. 5b) confirms the coexistence of Zn and S in the surface of CdS/Zn_1−x_Cd_x_S/ZnS css-NRs. The positions of Zn 2p_3/2_ and Zn 2p_1/2_ high resolution XPS signals for the CdS/Zn_1−x_Cd_x_S/ZnS sample (Fig. 5c) are located at 1022.5 and 1045.3 eV, respectively, which agree well with the values reported for the divalent zinc^36^. The single S 2p peak at 161.9 eV indicates that sulfur is present as a sulfur ion as shown in Fig. 5d^37^.

## Discussion

**Figure 6:**
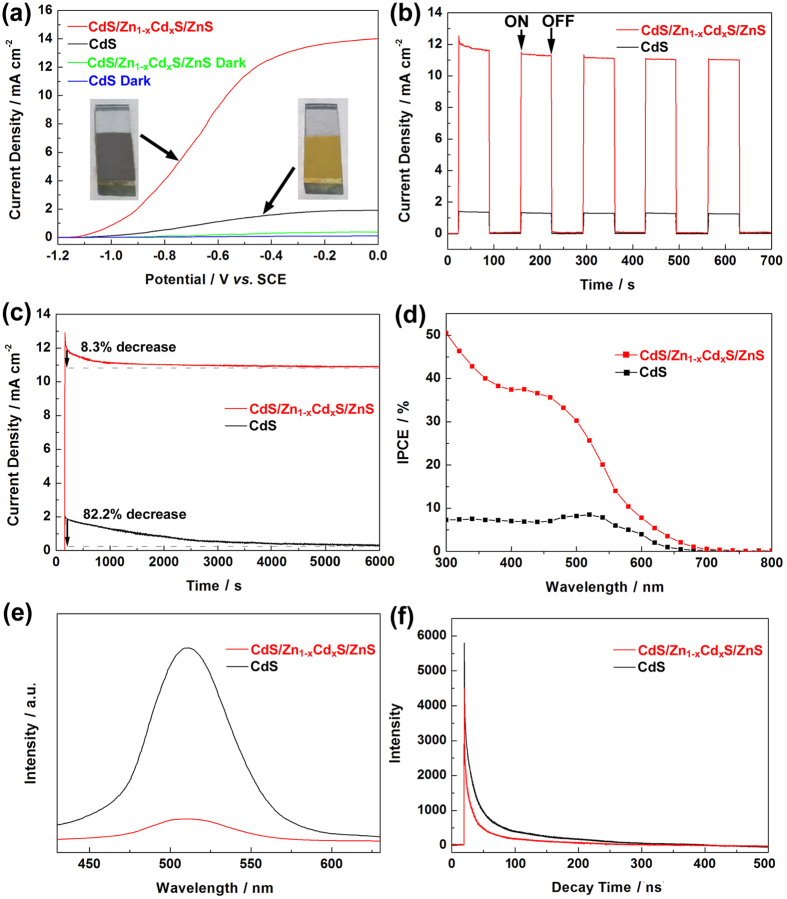
J-V profile over CdS and CdS/Zn_1−x_Cd_x_S/ZnS nanorod array photoanodes (**a**) and J-t of CdS and CdS/Zn_1−x_Cd_x_S/ZnS nanorod array photoanodes measured at −0.5 V versus SCE under AM 1.5G light (100 mW cm^−2^) with five on/off cycles (**b**). Stability test of CdS and CdS/Zn_1−x_Cd_x_S/ZnS nanorod array photoanodes under the illumination of AM 1.5G (**c**). IPCE spectra measured at an applied bias of −0.5 V vs. SCE (**d**). Photoluminescence (PL) spectra of CdS and CdS/Zn_1−x_Cd_x_S/ZnS photoanodes at an excitation wavelength of 405 nm (**e**) and time-resolved photoluminescence (TRPL) spectra of CdS and CdS/Zn_1−x_Cd_x_S/ZnS photoanodes monitored at the same excitation wavelength (**f** ).

Figure 6a shows the J-V curves of CdS and CdS/Zn_1−x_Cd_x_S/ZnS photoanodes obtained by linear sweep voltammetry measurements in a electrolyte containing Na_2_S (0.25 M) and Na_2_SO_3_ (0.35 M) using a three electrode electrochemical cell under simulated sunlight irradiation (AM 1.5G light of 100 mW cm^−2^). As shown in Fig. 6a, the CdS/Zn_1−x_Cd_x_S/ZnS photoanode shows the best performance with the photocurrent density at 0.0 V vs. saturated calomel electrode (SCE) of about 14.0 mA cm^−2^, nearly 8 times higher than that of bare CdS sample (1.8 mA cm^−2^). To the best of our knowledge, this is the highest photocurrent density obtained based on one-dimensional CdS nanoarchitectures^38^,^39^,^40^. Original CdS nanorod and CdS/Zn_1−x_Cd_x_S/ZnS css-NR photoanodes were further tested under chopped illumination of sunlight (100 mW cm^−2^) with an applied potential of −0.5 V vs. SCE. Two curves from Fig. 6b display fast and stable photoresponse feature (J-t curves) of the samples: the photocurrent increases steeply to a saturated value after light on and decays to the origin value once the illumination is switched off. The stable instantaneous current generation with a slight spike of photocurrent reveals the existence of an effective charge directional transport process among the css NRs^41^,^42^. From a practical application standpoint, the stabilities of CdS array and CdS/Zn_1−x_Cd_x_S/ZnS css-NR photoanodes were tested for prolonged period of 6000 s fixed potential of −0.5 V vs. SCE under AM 1.5G simulated. Particularly since CdS crystal is unstable due to the photocorrosion (CdS + 2h^+^ → Cd^2+^ + S)^43^. In contrast, the shells show a positive role in stabilizing the photocurrent against the photocorrosion. For single CdS sample, 82.2% reduction in the photocurrent is detected, while in css-NR sample, only 8.3% reduction is observed after the stability tests. Moreover, serious photocorrosion can be found from the exposed CdS nanorods after measurement of the stability (see Figures S3 and 4 in the Supporting Information). Incorporated the lamellar solid solution intermediate, the CdS/Zn_1−x_Cd_x_S/ZnS css-NRs sample exhibited a superb antiphotocorrosion ability over the complete 6000 s compared to our previous research (only 900 s, ZnS/CdS core/shell nanorods)^23^. The inherent improvement of stability can attributed to the growth of ultrathin intermediate layer which act as a tunneling junction for charge transport as well as the protection layer due to the low density of defect states of the two interfaces^22^. Neither the XRD patterns (see Figure S4 in the Supporting Information) nor the microstructure (see Figure S5 in the Supporting Information) of CdS/Zn_1−x_Cd_x_S/ZnS css-NRs exhibit distinct variations after 6000 s, further conforming the attractive PEC stability.

To further probe the origin of photocurrent generation, IPCE of the multilayered architecture as well as single CdS were investigated under −0.5 V bias as a function of incident light wavelength. In comparison to CdS electrode, the CdS/Zn_1−x_Cd_x_S/ZnS css-NRs electrode shows substantially enhanced IPCE values over the entire wavelength range of 300–800 nm, which is consistent with the J-V curves and UV-vis spectra. Apparently, CdS/Zn_1−x_Cd_x_S/ZnS exhibits a high IPCE of 51% at 300 nm, which is over 7 times higher than that of the uncoated CdS photoanode (7%). The enhancement of IPCE of the CdS/Zn_1−x_Cd_x_S/ZnS css-NR array electrode could be ascribed to enhanced light absorption as well as the efficient charge collection.

The separation and transfer mechanism of the photogenerated charge carriers for the drastically enhanced PEC performance was revealed by PL and time resolved PL (TRPL) spectra on the CdS/Zn_1−x_Cd_x_S/ZnS css-NRs as compared to the bare CdS nanorods (Fig. 6e,f). Figure 6e displays the PL curve at an excitation wavelength of 405 nm, and an obvious emission band at ~510 nm is found from CdS curve caused by the direct electron–hole recombination of band transition^44^. Incorporated the shells (ZnS and Zn_1−x_Cd_x_S), multilayered nanorods shows remarkably quenched characteristic, attributing to the efficient transfer of photogenerated charge carrier between core and shells, which can suppress the electron–hole recombination, and finally enhance the PEC activity and stability. The high-efficiency charge carriers transfer process in CdS/Zn_1−x_Cd_x_S/ZnS css-NRs has also been confirmed by the TRPL spectra as shown in Fig. 6f. The data showed the CdS nanorods have short lifetimes (828 ns), which is caused by the localized exciton recombination from de-trapping of carriers^45^. Compared to bare CdS, the PL lifetime is obviously shortened in the CdS/Zn_1−x_Cd_x_S/ZnS css-NRs which is 521 ns. Shorter lifetimes in TRPL could be the main influence on PEC test in our novel css-NR architectures because of the efficient charge transfer between core and shell across the interlayer smoothly and suppression of the electron–hole recombination.

**Figure 7:**
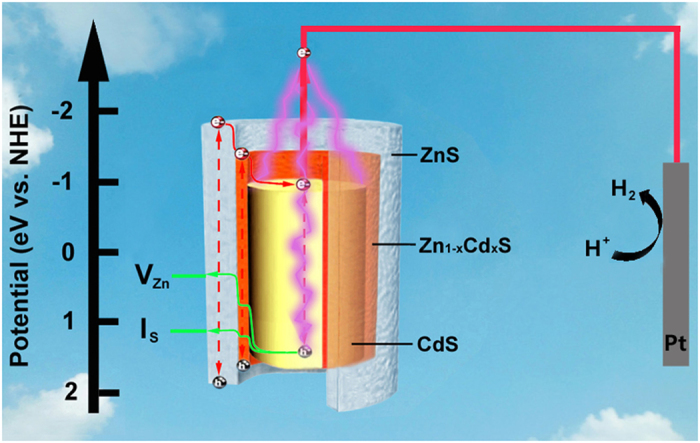
Schematic illustrating the energy band structure and charge transfer on CdS/Zn_1−x_Cd_x_S/ZnS css-NR under AM 1.5G illumination.

Based on the above results, a possible mechanism of PEC water splitting for the energy band structure and charge transfer is proposed in Fig. 7. Upon irradiation, photogenerated electron-hole pairs can be excited in the overall of the three components (dotted arrow). Due to the staggered gap and potential difference, the charge carries can efficient separate (V_Zn_ and I_s_ acceptor level^46^) through fast inverse transport in the as-prepared quasi-type II css architectures along the direction of the solid arrow as shown in Scheme 1. Different from the typical heterojunction, the lattice well matched lamellar growing on the interface of ZnS/CdS can act as a protection layer, as well as a tunneling junction for charge transport due to the two low density of defect states of the interfaces.

In summary, for the first time, the novel CdS/Zn_1−x_Cd_x_S/ZnS core/shell/shell nanorods (css-NRs) array photoanode have been successfully fabricated through a simple and efficient two-step aerosol assisted chemical vapor deposition (AACVD) approach. Thanks to the incorporation of the solid solution Zn_1−x_Cd_x_S transition layer, a maximum photocurrent intensity of the css-NRs photoanode increased to 14.0 mA cm^−2^ at a potential of 0.0 V vs. SCE under AM 1.5G illumination, which was nearly 8 times higher than that of the perfect CdS photoanode. The interlayer with lattice well matched can act as a bridge to smooth the band gap between the core (CdS) and outer shell (ZnS) due to the establishment of two low density of defect states of the interfaces in the unique one-dimensional nanoarchitectures. This work has revealed potential advantages of the core/shell structures with a tunneling junction interlayer for photoelectrochemical (PEC) water splitting application and opened a promising avenue for the design and fabrication of novel one-dimensional CdS-based core/shell heterojunction arrays for other electronic nanodevices.

## Methods

### Synthesis of cadmium and zinc diethyldithiocarbamate complex

Cadmium nitrate tetrahydrate (99%), zinc nitrate hexahydrate (99.99%), and sodium diethyldithiocarbamate (99%), were purchased from Aladdin and were used as received. Absolute ethanol (AR, Shanghai Chemical Factory of China) was used without further purification. The precursor cadmium diethyldithiocarbamate complex (Cd(S_2_CN(C_2_H_5_)_2_)_2_) was prepared from stoichiometric amounts of Cd(NO_3_)_2_ · 4H_2_O (1 mmol) and NaS_2_CN(C_2_H_5_)_2_ (2 mmol) in 60 mL of absolute ethanol. After stirred for 15 min, the yellow precipitate was collected by centrifugation and washed with absolute ethanol and distilled water four times, and finally vacuum-dried at room temperature. Precursor zinc diethyldithiocarbamate (Zn(S_2_CN(C_2_H_5_)_2_)_2_) was synthesized by the same method described for Cd(S_2_CN(C_2_H_5_)_2_)_2_ using Zn(NO_3_)_2_ · 6H_2_O (1 mmol) as zinc source. The reaction for the preparation of cadmium and zinc precursor can be formulated as follows:


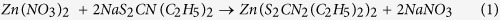



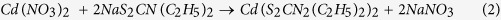


### Preparation of CdS and CdS/Zn_1−x_Cd_x_S/ZnS css-NR Arrays by AACVD

In a typical deposition process, 0.35 g of precursor cadmium diethyldithiocarbamate (Cd(S_2_CN(C_2_H_5_)_2_)_2_) was dissolved in 25 mL of CH_2_Cl_2_ in a two-necked round-bottomed flask (100 mL). Cleared FTO glass (1 × 2 cm) was placed inside the quartz tube of a furnace (CARBOLITE). The flask containing precursor solution was kept in a water bath (room temperature) above the piezoelectric modulator of a PARKOO ultrasonic humidifier (Model YDH803EB). Thus, the aerosol droplets of the precursor generated were transferred into the hot wall zone of the reactor by carrier gas (Ar). After deposit of CdS nanorod arrays at 400 °C for 1 h, the same process was carried out to coat the ZnS shell (0.35 g of Zn(S_2_CN(C_2_H_5_)_2_)_2_) at 450 °C and maintain the temperature for 1.5 h. Finally, the temperature was increased and kept constant at 600 °C for 5 h. The furnace was cooled to room temperature naturally, and the product was collected.

## Materials characterization

The morphologies and quantitative elemental analyses of the obtained CdS-based core/shell samples were characterized by TEM, EDX line scanning using a JEOL JEM-2100F field-emission electron microscope operated at 200 kV, and HRTEM, high-angle annular dark-field scanning TEM (HAADF-STEM) operated at 200 kV. UV-vis absorption spectra of the as-prepared samples were characterized on a UV3600-NIR-recording spectrophotometer at resolution of 2 nm (Shimadzu, Japan). The metal composition of the samples was estimated using ICP (Optima 5300DV). XPS were acquired on an ESCALAB 250 with Al Kα (hυ = 1486.6 eV) as the excitation source. XRD patterns were performed on a BRUKER D8 Advance X-diffractometer (Cu Kα radiation, 1.54056 Å). PL emission and PL decay spectra were recorded with a fluorescence spectrophotometer (Edinburgh Instruments, FLSP-920) at room temperature.

## PEC measurements

PEC measurements of the bare CdS and css-NR films were carried out in a three-electrode configuration^47^,^48^,^49^ (CHI610B Electrochemical Workstation, Shanghai Chenhua Instrument Co., Ltd., Shanghai, China) (obtained films → working electrode, Pt foil → counter electrode, and standard saturated calomel electrode (SCE) → reference electrode). The current density vs. potential (J-V) curves tested at a potential ranging from −1.2 to 0 V (vs. SCE), with a scan rate of 20 mV s^−1^. The J-t (time) curves are measured at the external bias of −0.5 V (vs. SCE). The electrolyte was an aqueous solution containing Na_2_S (0.25 M) and Na_2_SO_3_ (0.35 M). A 300W Xe lamp (91160, Newport, USA) was used as light source. An AM 1.5G filter was used to obtain one sun light intensity (100 mW cm^−2^). Another Xe lamp (300 W, CHF-XM-500, Changtuo Technology Co., Ltd.) and a monochromator (monochromator 300) were used to investigate the wavelength dependent photocurrent, and the output intensity of the light source was measured with the same radiometer (1916-R, Newport). The incident photon to electron conversion efficiency (IPCE) of the samples was calculated as follows:


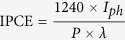


where *I*_ph_ is the photocurrent density (mA cm^−2^), *P* and *λ* are the incident light intensity (mW cm^−2^) and wavelength (nm), respectively.

## Additional Information

**How to cite this article**: Zhang, J. *et al*. Interface induce growth of intermediate layer for bandgap engineering insights into photoelectrochemical water splitting. *Sci. Rep.* **6**, 27241; doi: 10.1038/srep27241 (2016).

## Supplementary Material

Supplementary Information
