# Supplementary material for: Interface induce growth of intermediate layer for bandgap engineering insights into photoelectrochemical water splitting
Source: Sci Rep. 2016 Jun 2;6:27241. doi: 10.1038/srep27241 (PMC4890116; doi:10.1038/srep27241)
Supplement: Supplementary Information [file srep27241-s1.pdf]

Supplementary Information

Interface Induce Growth of Intermediate Layer for Bandgap Engineering Insights into Photoelectrochemical Water Splitting

Jian Zhang[1], Qiaoxia Zhang[1], Lianhui Wang[1], Xing'ao Li[1,]* and Wei Huang[1,2,]*

[1] Key Laboratory for Organic Electronics and Information Displays & Institute of Advanced Materials (IAM), Jiangsu National Synergetic Innovation Center for Advanced Materials (SICAM), Nanjing University of Posts & Telecommunications, Nanjing 210023, China.

[2] Key Laboratory of Flexible Electronics (KLOFE) & Institute of Advanced Materials (IAM), Jiangsu National Synergetic Innovation Center for Advanced Materials (SICAM), Nanjing Tech University (NanjingTech), Nanjing 211816, China.

***** lxahbmy@126.com; iamwhuang@njupt.edu.cn

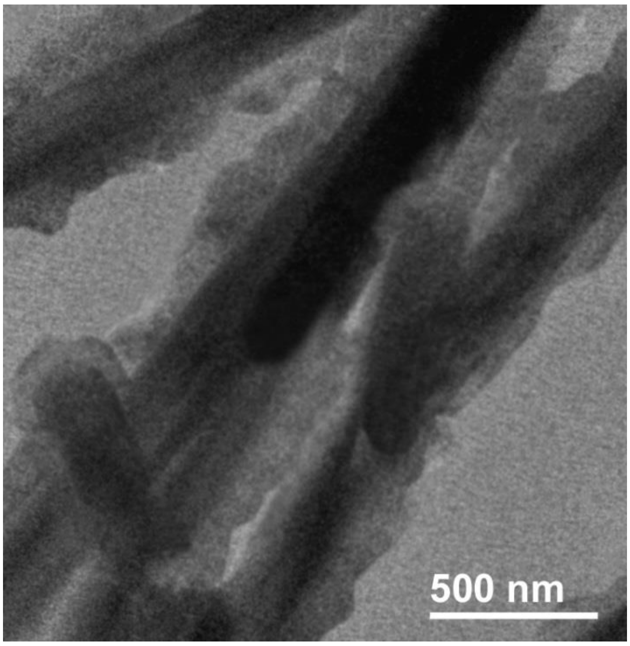

Figure S1. TEM image of CdS/ZnS core/shell nanorods.

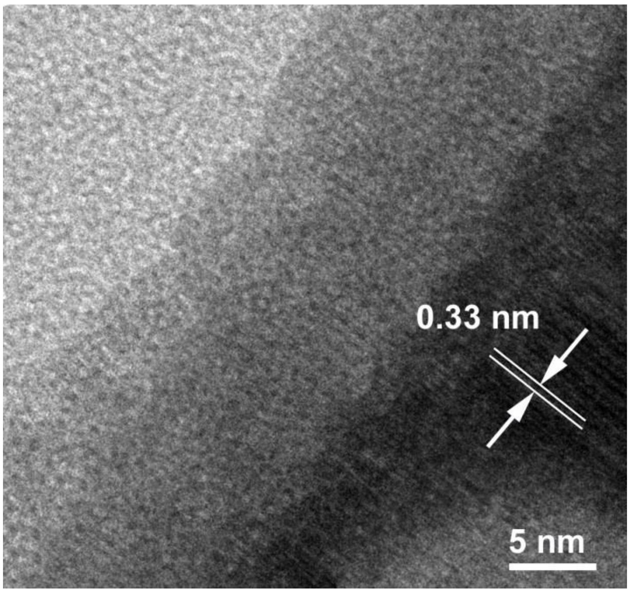

Figure S2. HRTEM image of CdS/ZnS core/shell nanorods.

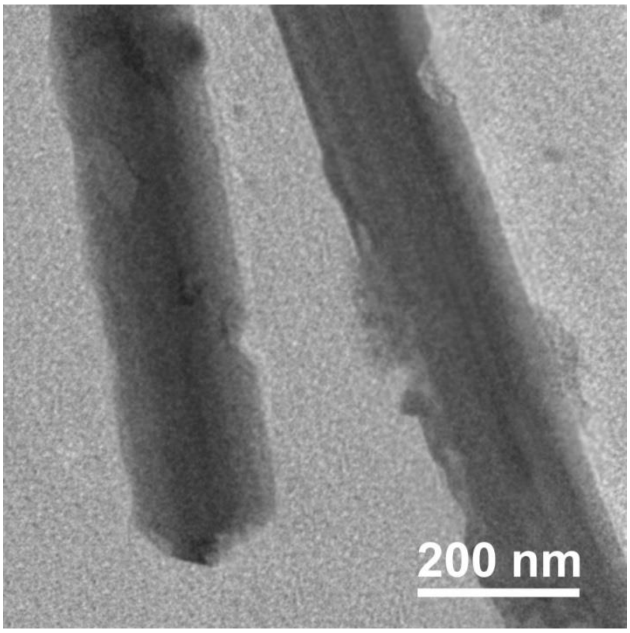

Figure S3. SEM image of the bare CdS nanorods after the stability test (6000 s).

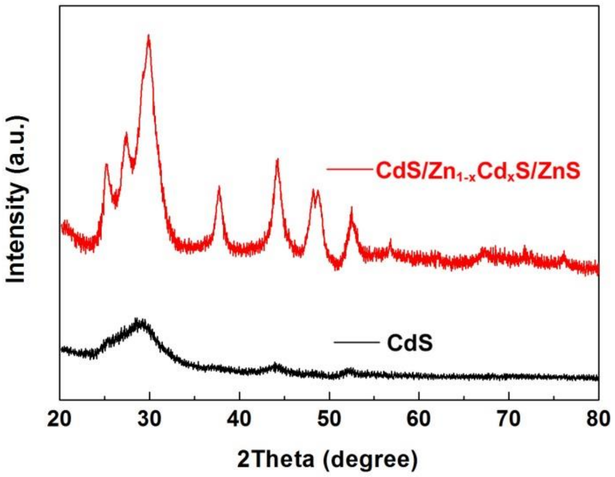

Figure S4. XRD patterns of the CdS and $CdS/Zn_{1-x}Cd_xS/ZnS$ nanorods after the 6000 s stability test.

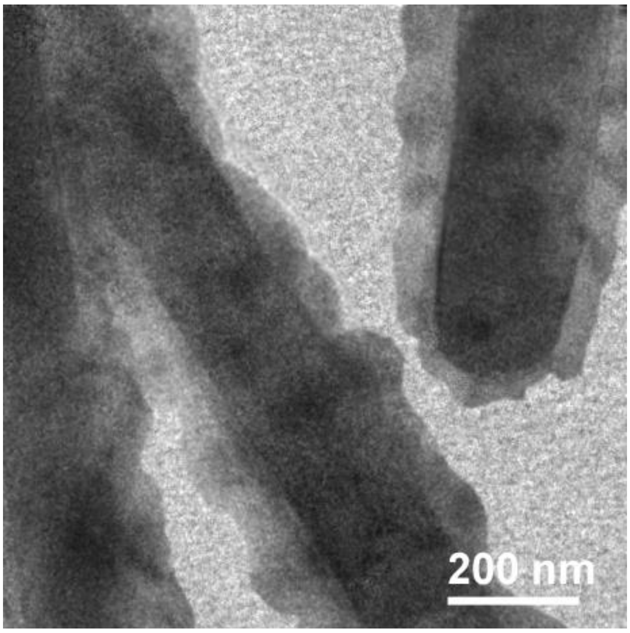

Figure S5. SEM image of the $CdS/Zn_{1-x}Cd_xS/ZnS$ nanorods after the stability test (6000 s).
